# Extracellular high‐mobility group box 1 mediates pressure overload‐induced cardiac hypertrophy and heart failure

**DOI:** 10.1111/jcmm.12743

**Published:** 2015-12-09

**Authors:** Lei Zhang, Ming Liu, Hong Jiang, Ying Yu, Peng Yu, Rui Tong, Jian Wu, Shuning Zhang, Kang Yao, Yunzeng Zou, Junbo Ge

**Affiliations:** ^1^ Department of CardiologyShanghai Institute of Cardiovascular DiseasesZhongshan HospitalShanghai Medical College of Fudan UniversityShanghaiChina

**Keywords:** high‐mobility group box 1, pressure overload, hypertrophy, heart failure

## Abstract

Inflammation plays a key role in pressure overload‐induced cardiac hypertrophy and heart failure, but the mechanisms have not been fully elucidated. High‐mobility group box 1 (HMGB1), which is increased in myocardium under pressure overload, may be involved in pressure overload‐induced cardiac injury. The objectives of this study are to determine the role of HMGB1 in cardiac hypertrophy and cardiac dysfunction under pressure overload. Pressure overload was imposed on the heart of male wild‐type mice by transverse aortic constriction (TAC), while recombinant HMGB1, HMGB1 box A (a competitive antagonist of HMGB1) or PBS was injected into the LV wall. Moreover, cardiac myocytes were cultured and given sustained mechanical stress. Transthoracic echocardiography was performed after the operation and sections for histological analyses were generated from paraffin‐embedded hearts. Relevant proteins and genes were detected. Cardiac HMGB1 expression was increased after TAC, which was accompanied by its translocation from nucleus to both cytoplasm and intercellular space. Exogenous HMGB1 aggravated TAC‐induced cardiac hypertrophy and cardiac dysfunction, as demonstrated by echocardiographic analyses, histological analyses and foetal cardiac genes detection. Nevertheless, the aforementioned pathological change induced by TAC could partially be reversed by HMGB1 inhibition. Consistent with the *in vivo* observations, mechanical stress evoked the release and synthesis of HMGB1 in cultured cardiac myocytes. This study indicates that the activated and up‐regulated HMGB1 in myocardium, which might partially be derived from cardiac myocytes under pressure overload, may be of crucial importance in pressure overload‐induced cardiac hypertrophy and cardiac dysfunction.

## Introduction

Cardiac hypertrophy, which may eventually trigger the transition to heart failure, occurs in many forms of clinical conditions, including hypertension and valvular heart disease 1, 2. Although it is widely accepted that inflammation including various cytokines and receptors are involved in the pathological process of pressure overload‐induced cardiac hypertrophy and cardiac dysfunction 3, 4, 5, the molecular mechanisms underlying the progression of cardiac hypertrophy and succedent heart failure have not yet been completely defined.

High‐mobility group box 1 (HMGB1), which exhibits diverse biologic functions depending on its cellular location, is a non‐histone DNA‐binding nuclear protein presenting in nearly all cell types 6. In the nucleus, HMGB1 functions in stabilizing nucleosome structure and facilitating gene transcription in nucleus 7, while extracellular HMGB1, released by activated inflammatory cells and/or necrotic cells, functions as a pro‐inflammatory cytokine 8. Studies have been performed concerning the role of HMGB1 in cardiovascular diseases, but yielding conflicting results. In rats with myocardial infarction, intramyocardially injection of exogenous HMGB1 attenuates cardiomyocyte hypertrophy in non‐infarcted segment and improves global cardiac function 9, while HMGB1 blockade by neutralizing anti‐HMGB1 antibody results in thinning and expansion of the infarct scar and marked hypertrophy of the non‐infarcted area 10.

Moreover, it has been reported that exogenous HMGB1 induces hypertrophy in cardiomyocytes *in vitro* 11, while overexpression of nuclear HMGB1 prevents pressure overload‐induced cardiac hypertrophy and heart failure *in vivo* 12. Combining these with the fact that HMGB1 expression is increased in myocardium under pressure overload, accompanying with its translocation from the nucleus to the cytoplasm 12, 13, the hypothesis seems reasonable that extracellular HMGB1 may be of crucial importance in pressure overload‐induced cardiac injury. The aim of this study was, therefore, to make the hypothesis testable.

## Materials and methods

### Experimental animals

Male wild‐type C57BL/6J mice (8–10 weeks old, 22–24 g, purchased from Shanghai Branch of National Rodent Laboratory Animal Resources, Shanghai, China) were enrolled in this study. Mice were housed at 24 ± 2°C under a 12:12‐hr light–dark cycle with *ad libitum* access to water and standard laboratory mouse chow. All experimental procedures in mice have been approved by the Animal Care and Use Committee of Zhongshan Hospital, Fudan University.

### Surgical interventions: transverse aortic constriction, proteins injection

Pressure overload was imposed on the heart of mice by transverse aortic constriction (TAC), as we described previously 14, 15. Recombinant HMGB1, HMGB1 box A (a competitive antagonist of HMGB1) (Hmgbiotech, Milano, Italy) or PBS was injected into the LV wall, as described previously 9, 16. Briefly, the mice were anaesthetized, endotracheally intubated and mechanically ventilated (type 7025; Harvard Apparatus, March‐Hugstetten, Germany). Partial left‐side thoracotomy to the second rib was performed and the sternum was retracted using a chest retractor. After the aortic arch was isolated, a blunted 27‐gauge needle was tied with a 7‐0 nylon suture to the aorta between the innominate and left common carotid arteries. The needle was immediately withdrawn after the ligation. Sham‐operated mice were subjected to identical interventions except for the constriction of the aorta. Before the ligation of the aortic arch, 200 ng of purified HMGB1 in 10 μl PBS solution containing rhodamine spheres was injected through a 32‐gauge needle. Five injections (2 μl per injection) were made in the LV wall (three injections in the anterior wall and two in the posterior wall). Successful injection was indicated by the presence of rhodamine in the site of injection. Control pressure overloaded mice were injected with either 200 ng of HMGB1 boxA, or PBS as mentioned above. For persistent inhibition of HMGB1, an additional injection of HMGB1 boxA was performed at 1 week after TAC.

### Cell culture and mechanical stretch of cardiac myocytes

Neonatal rat cardiac myocytes were prepared and given sustained mechanical stress, as we described previously 17, 18. Briefly, primary cultures of rat ventricular myocytes were obtained using 1 or 2‐day‐old Sprague‐Dawley rat pups, with the approval of the Institutional Animal Care and Use Committee of Fudan University. Cells were incubated in low glucose DMEM supplemented with 10% (v/v) Fetal Bovine Serum (FBS), 100 U/ml each of penicillin and streptomycin, and 20 mM HEPES (pH 7.2) at 37°C in humidified air with 5% CO_2_. Confluent monolayers exhibiting spontaneous contractions were developed in culture within 2 days. Cells were incubated in serum and antibiotic‐free conditions in silicon‐based plates pre‐coated with collagen for 24 hrs before sustained mechanical stretch to 120% of length for 2–24 hrs.

### Echocardiography measurement

Echocardiographic analyses were performed before (baseline) and at 2 and 4 weeks post TAC in mice, as we described previously 14, 19. Briefly, Transthoracic echocardiography was performed with an animal specific instrument (Vevo770; VisualSonics, Toronto, ON, Canada). After inhalation anaesthesia of isoflurane, two‐dimensional images were recorded in a parasternal long‐axis projection with guided M‐mode. The LV structure and systolic function were measured, including LV wall thickness, LV dimensions, LV volume. Percent fractional shortening (% LVFS) and ejection fraction (% LVEF) were calculated as described previously 20. All measurements were averaged for 3–5 consecutive cardiac cycles and were carried out by an experienced technician, who was unaware of the treatment of the mice.

### Invasive hemodynamic assessment

Aortic blood pressure (ABP) and LV pressure (LVP) were evaluated at 2 and 4 weeks after surgery, as we described previously 14, 19. Briefly, after anaesthesia, a micromanometer (Millar 1.4F, SPR 835; Millar Instruments, Houston, TX, USA) was inserted through the right common carotid artery into the aorta and carefully introduced into LV. The transducer was connected to a Power Laboratory system (AD Instruments, Castle Hill, NSW, Australia) and the end‐systolic ABP and end‐systolic LVP were obtained.

### Histological analyses

Mice were killed and hearts were excised at 2 and 4 weeks after TAC. Each heart was then excised, rinsed in normal saline, weighted, fixed in 10% formalin, embedded in paraffin and sectioned at 4‐μm thickness in the short axis at the papillary muscle level. The sections were stained with haematoxylin and eosin according to standard protocol. The cross‐sectional area (CSA) of cardiomyocytes was analysed quantitatively by morphometry of haematoxylin and eosin‐stained sections. Five random high‐power fields from each section were chosen and quantified. The original images were measured by using an automated image analysis system (Image‐Pro Plus 5.0; Media Cybernetics, Bethesda, MD, USA). Immunostaining for HMGB1 was performed on paraffin section. The primary antibody for HMGB1 (Abcam, Cambridge, UK) was incubated overnight before linked to secondary antibody (Invitrogen, Carlsbad, CA, USA). As a negative control, the primary antibody incubation was omitted in the procedure.

### Protein extraction and Western blot analyses

Total protein was extracted from homogenized LV tissues and cultured cardiac myocytes using RIPA lysis buffer (Beyotime, Nanjing, China), culture supernatants were concentrated using Amicon Ultra Centrifugal Filter Devices (Millipore, Billerica, MA, USA) according to the manufacturer's protocol. Proteins were electrophoresed in 12% polyacrylamide gel and transferred to a polyvinylidene fluoride membrane (Millipore). The protein expressions was detected by immuneblotting with antibody against HMGB1 (Abcam). After three washes, the blot was incubated with horse radish peroxidase‐conjugated rabbit secondary antibody immunoglobulin G (Kangchen Biotechnology, Shanghai, China). β‐actin (Kangchen Biotechnology) was used as the internal control. The antigen–antibody complexes were detected using Pierce ECL Western Blotting Substrate (Thermo Fisher Scientific, Rockford, IL, USA) and visualized densitometry was performed with LAS‐300 Image software (FUJIFILM, Kanagawa, Japan).

### RNA extraction and quantitative real‐time polymerase chain reaction

Total RNA was extracted from LV tissues using Trizol reagent (Invitrogen) according to the manufacturer's protocol. Real‐time PCR was performed with RT‐PCR kits (TaKaRa, Kyoto, Japan). Four micrograms of total RNA template were used to make cDNA by using AMV reverse transcriptase and random 9 mers as the first‐strand primer. Synthesized cDNA was used in qPCR experiments. Quantitative real‐time PCR was performed with a 40‐cycle two‐step PCR with sequence‐specific primer pairs under the ABI 7900 Fast RT‐PCR System (PE Applied Biosystems, Foster City, CA, USA). The expression level of each gene was normalized to that of β‐actin, which served as an endogenous internal control. Specific primers for atrial natriuretic peptide (ANP), brain natriuretic peptide (BNP) and β‐actin are mentioned in Table 1.

**Table 1:** Primer sequences for quantitative real‐time PCR

Gene	Forward (F) and reverse (R) primers
ANP	F: 5′‐TGGGACCCCTCCGATAGATC‐3′ R: 5′‐AGCGAGCAGAGCCCTCAGT‐3′
BNP	F: 5′‐CCTGGCCCATCGCTTCT‐3′ R: 5′‐CATCTGGGACAGCACCTTCA‐3′
β‐actin	F: 5′‐CGATGCCCTGAGGCTCTTT‐3′ R: 5′‐TGGATGCCACAGGATTCCA‐3′

ANP: atrial natriuretic peptide; BNP: brain natriuretic peptide.

### Statistical analyses

Data are presented as means ± S.E.M. Statistical differences were analysed by one‐way anova followed by multiple comparisons performed with post hoc least significant difference test using SPSS version 15.0 (SPSS Inc, Chicago, IL, USA). Values of *P* < 0.05 were considered statistically significant.

## Results

### TAC‐induced cardiac hypertrophy and LV dysfunction

**Figure 1:**
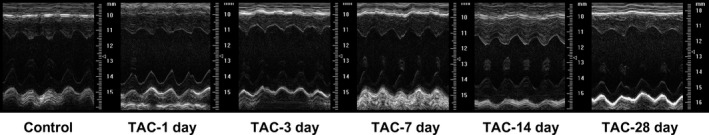
Representative M‐mode tracings of LV structure and LV function within 28 days after transverse aortic constriction (TAC).

**Figure 2:**
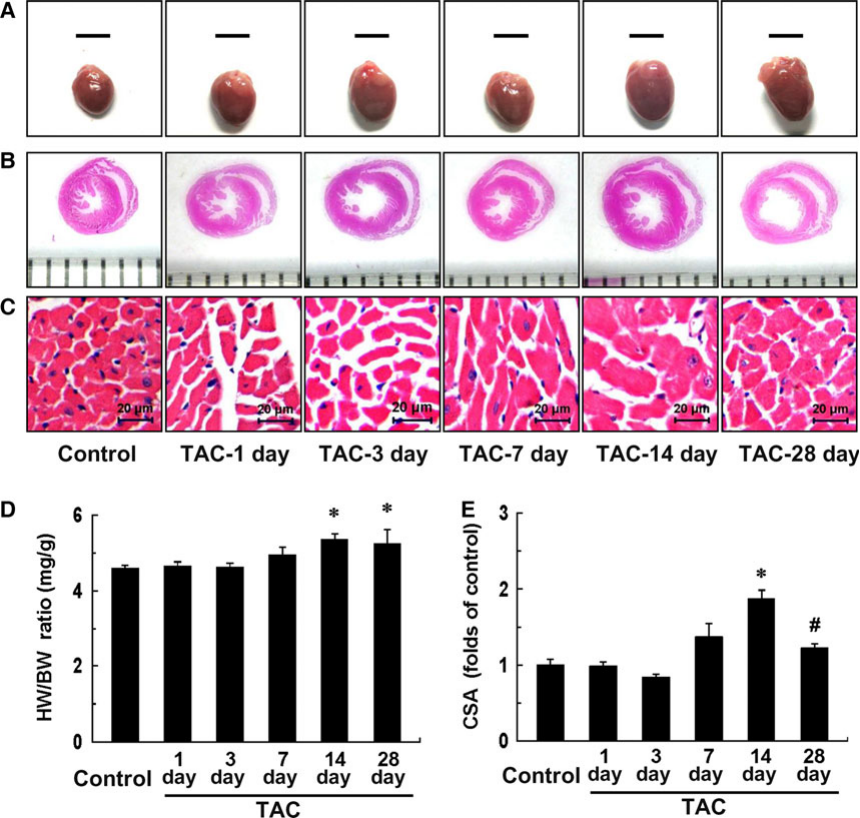
Transverse aortic constriction (TAC)‐induced cardiac hypertrophy. (**A**) Representative images of whole hearts within 4 weeks after TAC (scale bars, 5 mm). (**B** and **C**) Histological sections from hearts were stained with haematoxylin and eosin (**B**, grid size, 1 mm; **C**, scale bars, 20 μm). (**D**) Ratios of heart weight to bw (HW/BW). (**E**) Cross‐sectional area (CSA) of cardiomyocyte measured from five sections for one heart. Data represent the means ± S.E.M. *n* = 3–6 mice in each group. *P < 0.01 *versus* control group; ^#^ *P* < 0.05 *versus* TAC‐14d group.

Echocardiographic assessment showed that the LV function was temporarily impaired within 3 days post TAC (*P* < 0.01) and then recovered at 1 week, as indicated by the LV ejection fraction (LVEF). Nevertheless, LV dysfunction was observed again at 4 weeks after TAC (*P* < 0.01) (Fig. 1 and Table 2). During this pathological process, LV hypertrophy, increased cardiac mass as well as enlarged heart size and cardiomyocyte size were observed at 2 weeks post TAC (*P* < 0.01) (Figs 1 and 2 and Table 2). These results showed that pressure overload‐induced cardiac hypertrophy and LV dysfunction models in mice were successfully built by TAC.

**Table 2:** Echocardiography measurement and haemodynamic assessment in mice after TAC

Parameters	Control	TAC‐1d	TAC‐3d	TAC‐7d	TAC‐14d	TAC‐28d
HR (beats/min.)	516 ± 8	506 ± 11	496 ± 14	501 ± 13	501 ± 22	505 ± 13
LVEF (%)	74 ± 2	64 ± 2*	63 ± 2*	68 ± 2	71 ± 4	63 ± 2*
LVFS (%)	42 ± 2	34 ± 2*	33 ± 2*	38 ± 2	40 ± 3	33 ± 1*
End‐diastolic						
LVPW (mm)	0.58 ± 0.03	0.61 ± 0.05	0.65 ± 0.05	0.67 ± 0.02	0.85 ± 0.07*	0.68 ± 0.03
IVS (mm)	0.79 ± 0.02	0.80 ± 0.05	0.77 ± 0.02	0.82 ± 0.03	0.98 ± 0.04*	0.82 ± 0.05
LVID (mm)	3.42 ± 0.07	3.46 ± 0.10	3.71 ± 0.08†	3.60 ± 0.18	3.56 ± 0.03	3.90 ± 0.15*
LVV (μl)	46 ± 2	47 ± 4	56 ± 3*	52 ± 2	51 ± 1	62 ± 5*
End‐systolic						
LVPW (mm)	1.04 ± 0.02	1.06 ± 0.05	1.05 ± 0.05	1.08 ± 0.04	1.22 ± 0.04*	1.05 ± 0.06
IVS (mm)	1.08 ± 0.02	1.04 ± 0.05	1.05 ± 0.04	1.12 ± 0.05	1.26 ± 0.04*	1.08 ± 0.07
LVID (mm)	2.09 ± 0.07	2.44 ± 0.13†	2.59 ± 0.08*	2.33 ± 0.12	2.32 ± 0.16	2.82 ± 0.14*
LVV (μl)	12 ± 1	17 ± 2	21 ± 2*	16 ± 2	15 ± 3	24 ± 2*
ABP (mmHg)	105 ± 3	172 ± 6*	176 ± 7*	169 ± 5*	176 ± 9*	172 ± 8*
LVP (mmHg)	111 ± 3	175 ± 5*	179 ± 6*	172 ± 3*	181 ± 9*	176 ± 8*

*P < 0.01, ^†^ *P* < 0.05 *versus* control (n = 3 to 6 per group).

Values are expressed as mean ± S.E.M.

TAC: transverse aortic constriction; HR: heart rate; LVEF: LV ejection fraction; LVFS: LV fractional shortening; LVPW: left ventricle posterior wall thickness; IVS: interventricular septal wall thickness; LVID: LV internal dimension; LVV: LV volume; ABP: aortic blood pressure; LVP: LV pressure.

### TAC induced the translocation and expression of HMGB1 in myocardium

**Figure 3:**
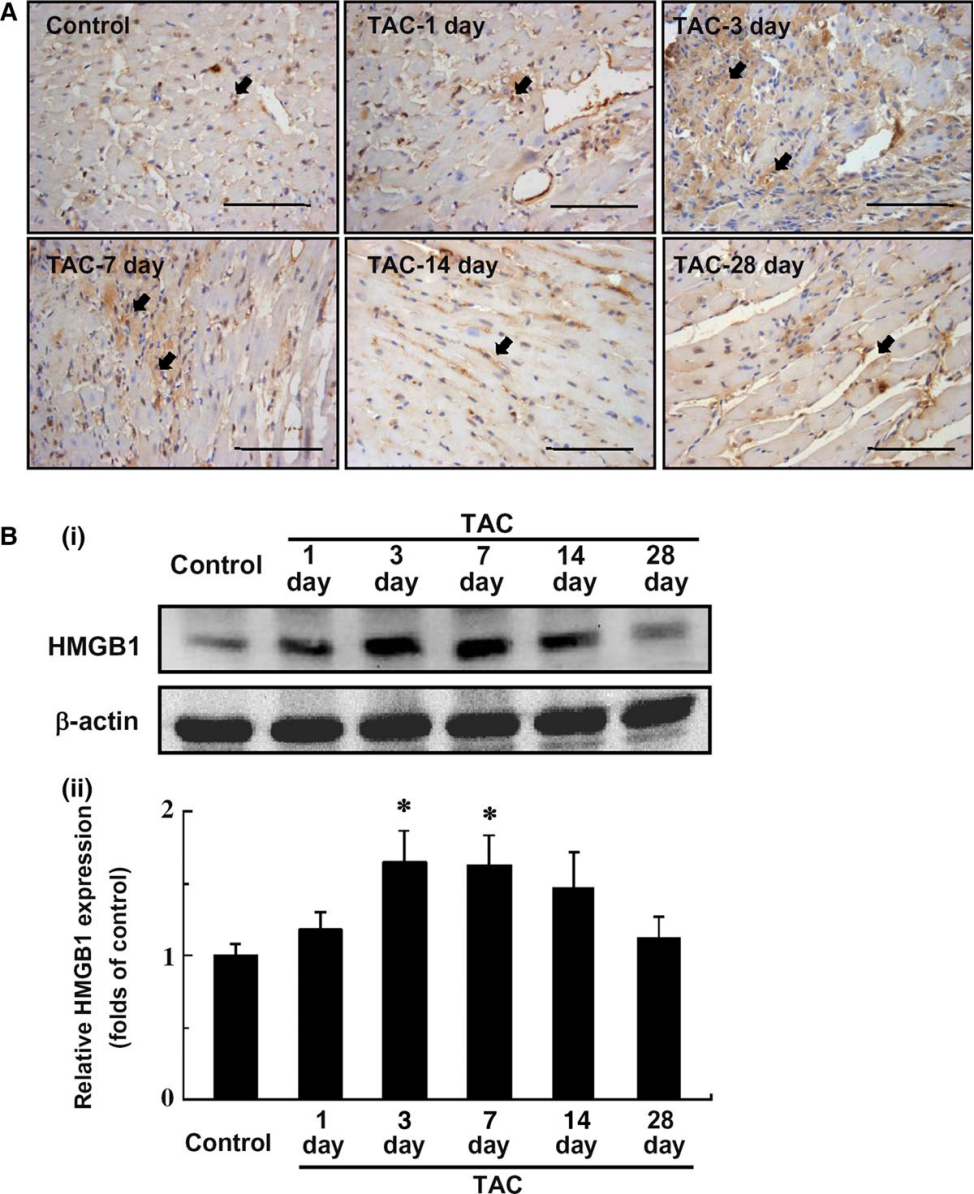
HMGB1 expression in myocardium after transverse aortic constriction (TAC). (**A**) Immunohistochemical staining of HMGB1 expression in the myocardium within 4 weeks after TAC. Blue, counterstaining of nuclei; Brown, HMGB1‐positive cells. Arrows indicate HMGB1‐positive cells (scale bars, 100 μm). Images are representative heart sections from 3 to 6 mice per group. (**B**) Western blot analysis of HMGB1 expression in the myocardium after TAC. (**Bi**) Representative photograms from four independent experiments are shown. (**Bii**) Quantitative analysis of four individual experiments. Relative expression of HMGB1 was normalized to β‐actin. HMGB1, high‐mobility group box‐1. Data represent the means ± S.E.M. *P < 0.05 *versus* control.

To determine whether HMGB1 is involved in TAC‐induced cardiac hypertrophy and LV dysfunction, the expression of HMGB1 were measured in myocardium at given time‐points after TAC (Fig. 3). Although the control mice showed only moderate nuclear staining for HMGB1 in myocardium, cardiac sections from mice undergoing TAC injury, especially the ones after 3 and 7 days of TAC, presented with significant translocation of HMGB1 from nucleus to both cytoplasm and intercellular space (Fig. 3A). Moreover, the number of HMGB1‐positive cells in myocardium at 3 and 7 days post TAC was increased compared with control, which might be accounted for by the infiltrating leucocytes (Fig. 3A). Additionally, Western blot analyses were performed on LV tissue lysates (Fig. 3Bi), and the results showed that HMGB1 expression was significantly increased after 3 days of TAC and remained increased within 1 week (*P* < 0.01) (Fig. 3Bii).

### Mechanical stress increased the expression and release of HMGB1 in cultured cardiomyocytes

**Figure 4:**
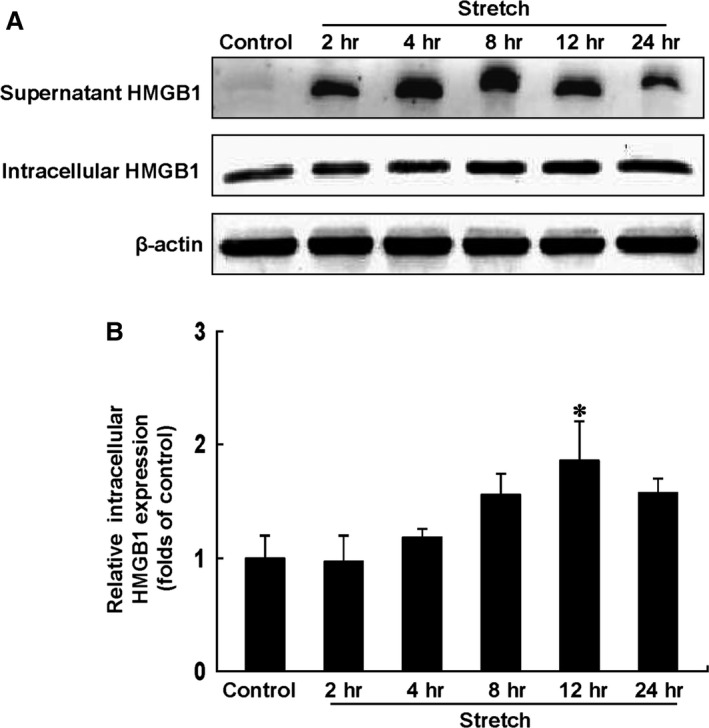
Western blot analysis of supernatant and intracellular HMGB1 expression in cultured cardiomyocytes under mechanical stress. (**A**) Representative photograms from four independent experiments are shown. (**B**) Quantitative analysis of four individual experiments. Relative expression of intracellular HMGB1 was normalized to β‐actin. HMGB1 indicates high‐mobility group box‐1. Data represent the means ± S.E.M. *P < 0.05 *versus* control.

Considering that the mechanical stress on myocardium is produced by TAC, we investigated the effects of mechanical stress on the expression and release of HMGB1 in isolated neonatal cardiomyocytes (Fig. 4). Consistent with the *in vivo* observations, although the supernatant expression of HMGB1 could hardly been seen under normal condition, mechanical stress immediately evoked the release of HMGB1 at 2 hrs and remained up to 24 hrs (Fig. 4A). Meanwhile, isolated cardiomyocytes showed a time‐dependent increase in intracellular HMGB1 protein expression that peaked after 12 hrs of mechanical stress (*P* < 0.05) (Fig. 4B).

### Effects of exogenous HMGB1 and HMGB1 boxA on TAC‐induced cardiac hypertrophy and LV dysfunction

**Figure 5:**
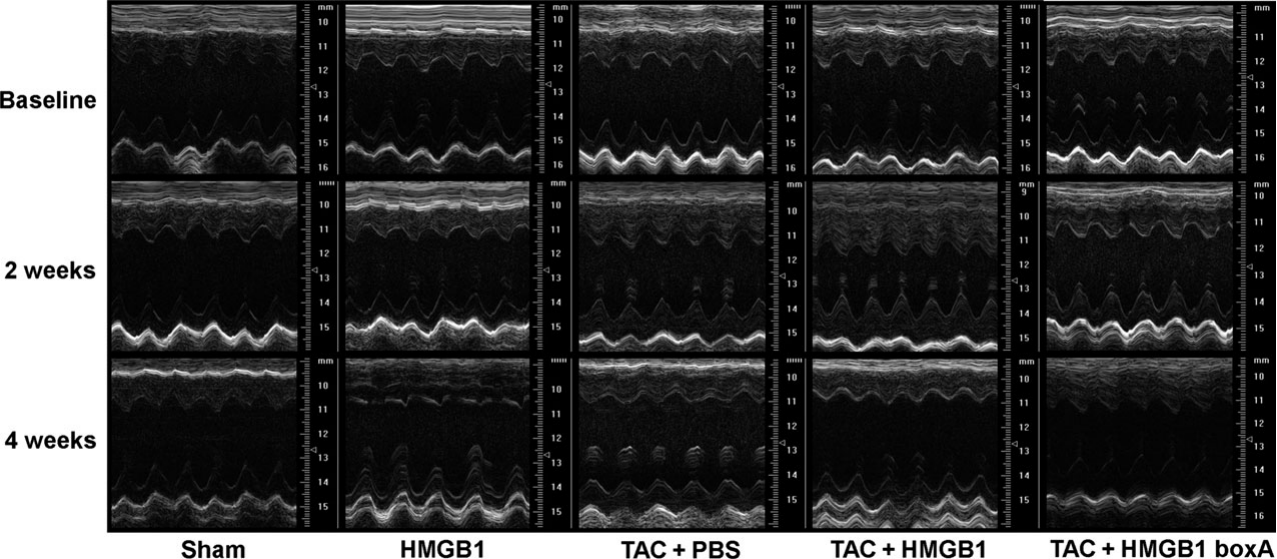
Representative M‐mode tracings of LV structure and LV function at baseline, 2 and 4 weeks after intervention. TAC: transverse aortic constriction; HMGB1: high‐mobility group box‐1; HMGB1 boxA: a competitive antagonist of HMGB1; PBS, phosphate buffered saline.

To examine the role of extracellular HMGB1 in the development of pressure overload‐induced cardiac hypertrophy and heart failure, exogenous HMGB1 and HMGB1 box A were used to treat mice with TAC. As shown in Figure 5 and Table 3, no difference in echocardiographic data was observed among all groups before the operation, while ABP and LVP at systole were similarly elevated at different time‐points after TAC. Moreover, TAC‐induced cardiac hypertrophy was observed at 2 weeks after TAC, which was significantly enhanced by exogenous HMGB1 overexpression in myocardium, as indicated by interventricular septal wall (IVS) thickness and left ventricle posterior wall (LVPW) thickness at both systole and diastole (*P* < 0.05). Contrarily, the increased IVS thickness was markedly attenuated by cardiac injection of HMGB1 box A in mice with TAC (*P* < 0.05). Similarly, HMGB1 box A tended to be associated, albeit non‐significantly, with the reduction in LVPW thickness. Meanwhile, temporary increased LVEF and LV fractional shortening (LVFS) were observed in TAC mice treated with HMGB1, which may be accounted for by the higher extent of cardiac hypertrophy. Additionally, overexpression of HMGB1 in myocardium alone had no impact on LV structure and function within 2 weeks.

**Table 3:** Echocardiography measurement and haemodynamic assessment in mice at 2 and 4 weeks after TAC

Parameters	Baseline					2 weeks					4 weeks				
	Sham	HMGB1	TAC+PBS	TAC+HMGB1	TAC+HMGB1 box A	Sham	HMGB1	TAC+PBS	TAC+HMGB1	TAC+HMGB1 box A	Sham	HMGB1	TAC+PBS	TAC+HMGB1	TAC+HMGB1 box A
HR (beats/min.)	496 ± 10	509 ± 3	501 ± 8	496 ± 6	506 ± 4	511 ± 6	499 ± 11	504 ± 5	500 ± 6	503 ± 4	504 ± 12	506 ± 10	507 ± 7	503 ± 9	506 ± 10
LVEF (%)	70 ± 2	70 ± 2	70 ± 1	69 ± 1	72 ± 1	71 ± 1	71 ± 2	72 ± 3	79 ± 3* ^,^ †	71 ± 3	70 ± 2	59 ± 1*	62 ± 2*	54 ± 4* ^,^ †	68 ± 3‡
LVFS (%)	39 ± 2	39 ± 2	39 ± 1	38 ± 1	41 ± 2	40 ± 1	40 ± 2	40 ± 2	48 ± 2* ^,^ †	40 ± 2	39 ± 2	30 ± 1*	33 ± 1*	28 ± 2* ^,^ †	37 ± 2§
End‐diastolic															
LVPW (mm)	0.68 ± 0.02	0.69 ± 0.03	0.73 ± 0.02	0.67 ± 0.02	0.68 ± 0.03	0.67 ± 0.02	0.73 ± 0.02	0.81 ± 0.03*	0.93 ± 0.04* ^,^ †	0.76 ± 0.02¶	0.69 ± 0.03	0.66 ± 0.04	0.77 ± 0.04	0.77 ± 0.04	1.00 ± 0.05* ^,^ †
IVS (mm)	0.77 ± 0.02	0.75 ± 0.02	0.76 ± 0.03	0.72 ± 0.02	0.75 ± 0.03	0.81 ± 0.04	0.78 ± 0.01	0.95 ± 0.02*	1.10 ± 0.05* ^,^ †	0.82 ± 0.05†	0.75 ± 0.04	0.69 ± 0.05	0.80 ± 0.03	0.79 ± 0.05	0.95 ± 0.06* ^,^ †
LVID (mm)	3.54 ± 0.04	3.59 ± 0.05	3.43 ± 0.06	3.56 ± 0.09	3.49 ± 0.09	3.44 ± 0.09	3.42 ± 0.13	3.31 ± 0.07	3.20 ± 0.09	3.42 ± 0.12	3.54 ± 0.11	3.79 ± 0.10	3.78 ± 0.08	3.94 ± 0.17*	3.62 ± 0.10
LVV (μl)	52 ± 2	53 ± 2	50 ± 2	53 ± 3	50 ± 2	51 ± 3	49 ± 4	46 ± 3	41 ± 3*	47 ± 3	52 ± 3	58 ± 4	59 ± 2	65 ± 7*	56 ± 4
End‐systolic															
LVPW (mm)	0.97 ± 0.03	0.99 ± 0.04	0.97 ± 0.03	0.94 ± 0.04	0.96 ± 0.03	0.99 ± 0.04	1.03 ± 0.05	1.18 ± 0.04*	1.35 ± 0.05* ^,^ †	1.08 ± 0.04**	0.99 ± 0.03	0.83 ± 0.06*	1.09 ± 0.05	1.06 ± 0.06	1.30 ± 0.04* ^,^ †
IVS (mm)	1.02 ± 0.02	1.02 ± 0.03	1.01 ± 0.03	1.02 ± 0.02	0.99 ± 0.03	1.06 ± 0.03	1.09 ± 0.06	1.23 ± 0.02*	1.43 ± 0.05* ^,^ †	1.07 ± 0.03†	1.05 ± 0.05	0.91 ± 0.04* ^,^ †	1.05 ± 0.04	1.01 ± 0.04	1.18 ± 0.05* ^,^ †
LVID (mm)	2.38 ± 0.07	2.38 ± 0.07	2.34 ± 0.07	2.44 ± 0.08	2.33 ± 0.08	2.25 ± 0.09	2.17 ± 0.12	2.20 ± 0.11	1.99 ± 0.10	2.34 ± 0.17	2.37 ± 0.10	2.83 ± 0.10*	2.79 ± 0.08*	2.99 ± 0.23*	2.56 ± 0.16
LVV (μl)	16 ± 1	16 ± 1	15 ± 1	17 ± 1	14 ± 1	15 ± 1	14 ± 2	13 ± 2	9 ± 2	14 ± 2	16 ± 1	24 ± 2*	23 ± 2*	31 ± 7* ^,^ †	19 ± 3
ABP (mmHg)	NA	NA	NA	NA	NA	111 ± 5	113 ± 4	179 ± 5*	193 ± 4*	178 ± 7*	111 ± 3	114 ± 3	183 ± 13*	183 ± 16*	191 ± 9*
LVP (mmHg)	NA	NA	NA	NA	NA	116 ± 4	114 ± 4	181 ± 7*	201 ± 2*	180 ± 6*	115 ± 4	115 ± 3	184 ± 14*	188 ± 15*	192 ± 10*

*P < 0.05 *versus* respective Sham group, ^†^ *P* < 0.05 *versus* respective TAC+PBS group, ^‡^ *P* = 0.10, ^§^ *P* = 0.12, ^¶^ *P* = 0.20, **P = 0.12 *versus* respective TAC+PBS group (*n* ≥ 6 per group).

Values are expressed as mean ± S.E.M.

TAC: transverse aortic constriction; HMGB1: high‐mobility group box‐1; HR: heart rate; LVEF: LV ejection fraction; LVFS: LV fractional shortening; LVPW: left ventricle posterior wall thickness; IVS: interventricular septal wall thickness; LVID: LV internal dimension; LVV: LV volume; ABP: aortic blood pressure; LVP: LV pressure; NA: not available.

With the reverse of cardiac hypertrophy, LV dysfunction and enlargement, which were indicated by LVFS, LVEF, LV internal dimension (LVID) and LV volume (LVV) were observed at 4 weeks after TAC, as compared with the sham‐operated mice (*P* < 0.05) (Fig. 5 and Table 3). Moreover, the impairment of LV function induced by TAC was deteriorated by exogenous HMGB1 overexpression, as shown in the further reduction of LVEF and LVFS (*P* < 0.05). Meanwhile, cardiac hypertrophy was observed in mice treated with HMGB1 box A at 4 weeks after TAC, which was accompanied by the trend of preservation of cardiac systolic function, as compared with the controls. Furthermore, overexpression of HMGB1 in myocardium alone resulted not only in the decrease of IVS and LVPW thickness, but also in reduction of LVEF and LVFS at 4 weeks after the intervention (*P* < 0.05) (Fig. 5 and Table 3).

**Figure 6:**
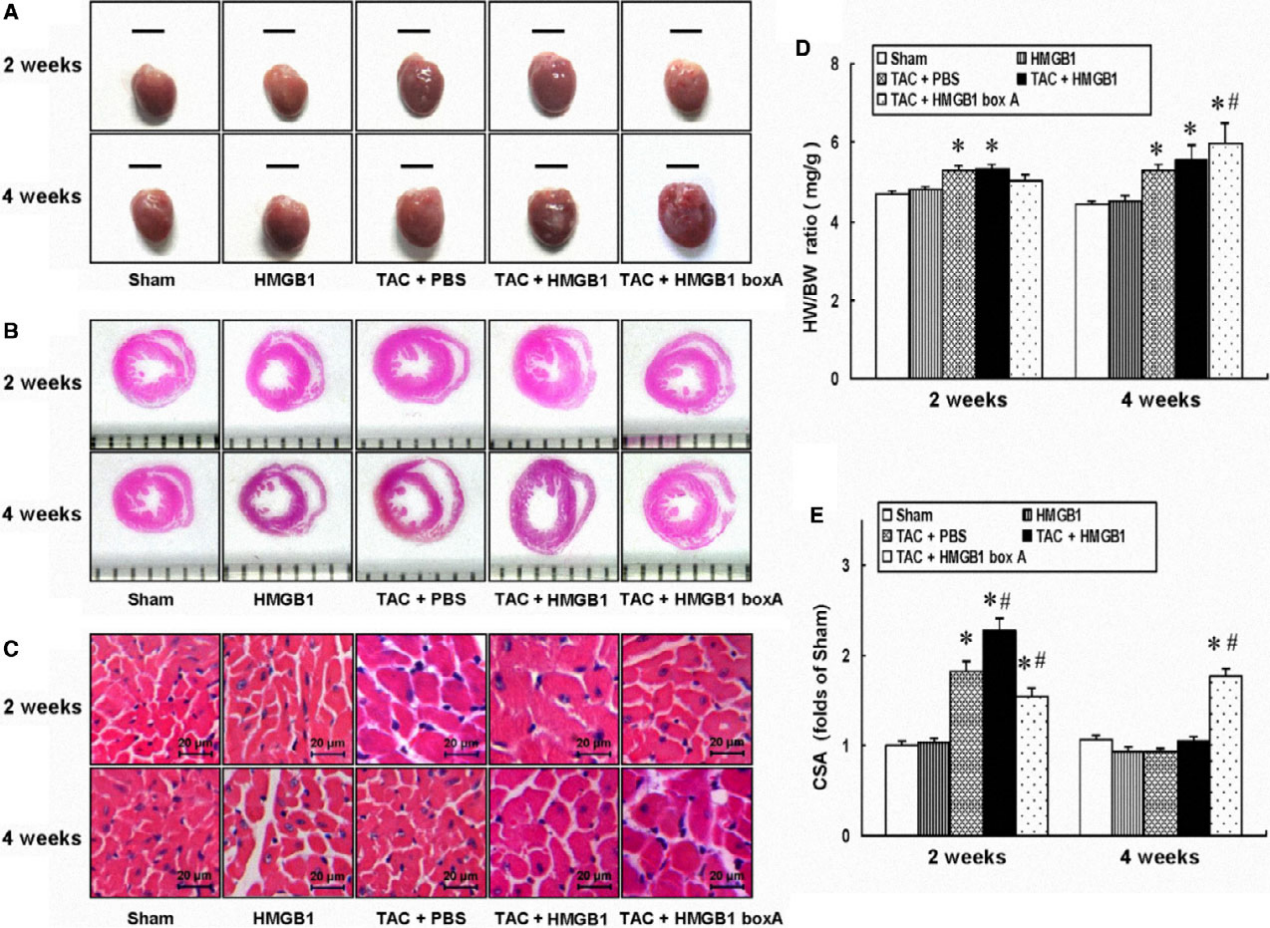
Effects of exogenous HMGB1 and HMGB1 box A on TAC‐induced cardiac remodelling at 2 and 4 weeks. (**A**) Representative images of whole hearts at in each group (scale bars, 5 mm). (**B** and **C**) Histological sections from hearts were stained with haematoxylin and eosin (**B**, grid size, 1 mm; **C**, scale bars, 20 μm). (**D**) Ratios of heart weight to bw (HW/BW). (**E**) Cross‐sectional area (CSA) of cardiomyocyte measured from five sections for one heart. TAC: transverse aortic constriction; HMGB1: high‐mobility group box‐1; HMGB1 boxA: a competitive antagonist of HMGB1; PBS, phosphate buffered saline. Data represent the means ± S.E.M. *n* ≥ 6 mice in each group. *P < 0.05 *versus* sham group; ^#^ *P* < 0.05 *versus* respective TAC+PBS group.

To verify the aforementioned cardiac remodelling, more analyses were performed. As shown in Figure 6, the ratio of heart weight (HW) to BW was increased in mice with TAC at 2 weeks after the operation, except for the ones simultaneously be treated by HMGB1 box A, while the ratio was up‐regulated in all mice with TAC at 4 weeks (Fig. 6D), which was in accordance with the results of echocardiographic analyses. Moreover, CSA of cardiomyocytes were measured in mice with different treatment, and the results showed that CSA was increased in mice at 2 weeks after TAC, which was enhanced by exogenous HMGB1 treatment and attenuated by HMGB1 box A injection in myocardium, while it was up‐regulated only in TAC mice treated by HMGB1 box A at 4 weeks (Fig. 6C and E).

**Figure 7:**
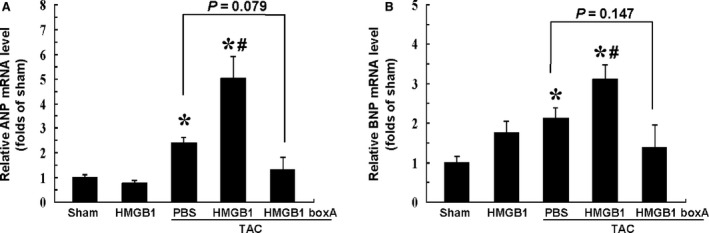
Quantitative Real‐Time PCR analyses of relevant mRNA expression in myocardium at 2 weeks after intervention. (**A**) Expression of ANP mRNA in each group. (**B**) Expression of BNP mRNA in each group. β‐actin served as internal control. ANP: atrial natriuretic peptide; BNP: brain natriuretic peptide; TAC: transverse aortic constriction; HMGB1: high‐mobility group box‐1; HMGB1 boxA: a competitive antagonist of HMGB1; PBS, phosphate buffered saline. Data represent the means ± S.E.M. of four independent experiments. *P < 0.05 *versus* sham group; ^#^ *P* < 0.05 *versus* respective TAC+PBS group.

Relevant foetal cardiac genes expression were further measured at 2 weeks after intervention. The results showed that the expressions of *ANP* and *BNP* were up‐regulated by more than twofold in mice with TAC, as compared with the sham‐operated ones (*P* < 0.05), which was enhanced by cardiac HMGB1 overexpression (*P* < 0.05), and tended to be, albeit non‐significantly, attenuated by HMGB1 box A (Fig. 7).

## Discussion

Evidence suggest that inflammation is involved in the progression of pressure overload‐induced cardiac hypertrophy and heart failure, but the mechanisms have not yet been fully characterized 3, 4, 5. In the present study, we addressed the potential contribution of local HMGB1 in pressure overload‐induced cardiac hypertrophy and LV dysfunction.

High‐mobility group box 1 was initially identified as a nuclear protein that regulates various transcriptional factors to stabilize the nucleosome 21, while extracellular HMGB1, which can be either passively released from necrotic cells or actively secreted by activated inflammatory cells and stressed cells 8, 22, is reported to modulate inflammation through its high affinity receptors on the surface of target cells, including the receptor for advanced glycation end products and toll‐like receptors 2 and 4 23, 24. There exist growing recognition and experimental evidence to support that HMGB1 plays a pivotal role not only in the diseases such as sepsis, autoimmune disease, acute hepatic necrosis, acute lung injury 25, 26, 27, 28 but also in various heart diseases, including myocardial infarction and ischaemia‐reperfusion injury, however, with no consensus on the function of HMGB1 on the pathogenesis of the diseases 9, 16, 29, 30, 31, 32, 33, 34.

In line with previous studies 12, 13, we showed that cardiac HMGB1 expression was increased during the pathological process of pressure overload‐induced cardiac hypertrophy, accompanying with its translocation from the nucleus to the cytoplasm. The translocation and increased expression of HMGB1 protein suggest a combined expression pattern consisting of release and new synthesis of HMGB1. Moreover, as one type of the major cells existing in myocardium, cardiac myocytes may act as ‘inflammatory cells’ under various stimuli, releasing cytokines such as tumour necrosis factor‐alpha and interleukin‐6 35, 36. Likewise, we have previously found that oxidative stress not only elevated intracellular and extracellular HMGB1 expression in isolated cardiac myocytes but also induced its translocation from nucleus to cytoplasm 37. Consistent with this notion, we have found in this study that mechanical stress triggered the release of HMGB1 from isolated cardiac myocytes, along with an increase of intracellular HMGB1 expression in a time‐dependent manner, indicating that the elevated HMGB1 protein in myocardium under pressure overload may be derived, at least partially, from cardiac myocytes.

Recently, it has been reported that exogenous HMGB1 induces hypertrophy in isolated cardiac myocytes 11, while overexpression of nuclear HMGB1 prevents pressure overload‐induced cardiac hypertrophy and heart failure 12. Considering that extracellular HMGB1 exerts diverse functions *via* different receptors in various diseases, it seems reasonable that the up‐regulated extracellular HMGB1 may be involved in pressure overload‐induced cardiac remodelling. As expected, we found that exogenous HMGB1 overexpression in myocardium by intramyocardially injection significantly enhanced pressure overload‐induced cardiac hypertrophy, while blockade of HMGB1 by HMGB1 box A tended to reverse the hypertrophy induced by pressure overload, which may be accounted for by the attenuation of local inflammation and relevant pathological changes.

Cardiac hypertrophy is considered to be an initially expected response of the heart to pressure overload, compensating for the increase in workload. In the long‐term, however, the initial response may become maladaptive, which is characterized by progressive LV dilation with an inadequate cardiac pumping activity 38, 39. We found in this study that instead of cardiac hypertrophy, decreased LVEF was observed in mice at 4 weeks after TAC, and exogenous HMGB1 overexpression further aggravated the impairment of cardiac function from this perspective. Nevertheless, cardiac hypertrophy was remained in TAC mice treated by HMGB1 box A at the same time, accompanied by the trend of preservation of LV systolic function, indicating that HMGB1 may be of crucial importance in TAC‐induced cardiac injury, and heart failure induced by pressure overload may be postponed by local HMGB1 blocking.

Moreover, previous studies have shown the negative inotropic effects of HMGB1 on both isolated contracting feline cardiac myocytes and left ventricle in isolated rat heart model of septic shock 40, 41. In the present study, overexpression of HMGB1 in myocardium alone failed to induce cardiac hypertrophy, but finally resulted not only in the decrease of LV thickness, but in the impairment of LV systolic function, suggesting that HMGB1 may act as a myocardial depressant factor during cardiac injury.

Taken together, this study showed that mice with TAC displayed marked LV hypertrophy, cardiac dysfunction as well as up‐regulation and translocation of HMGB1 expression in myocardium, which may partially be cardiac myocytes‐derived. Moreover, exogenous HMGB1 overexpression in myocardium not only promoted pressure overload‐induced cardiac hypertrophy but also aggravated LV systolic dysfunction in the long‐term, which may be reversed by HMGB1 box A, a competitive antagonist of HMGB1. Additionally, overexpression of HMGB1 alone did not trigger cardiac hypertrophy, but lead to LV dysfunction, along with the decrease in LV thickness. Nevertheless, further studies are needed to clarify the underling mechanisms of these pathological changes.

## Conflicts of interest

The authors confirm that there are no conflicts of interest.
